# Identification of essential residues for binding and activation in the human 5-HT_7(a)_ serotonin receptor by molecular modeling and site-directed mutagenesis

**DOI:** 10.3389/fnbeh.2015.00092

**Published:** 2015-05-08

**Authors:** Agata Antonina Rita Impellizzeri, Matteo Pappalardo, Livia Basile, Ornella Manfra, Kjetil Wessel Andressen, Kurt Allen Krobert, Angela Messina, Finn Olav Levy, Salvatore Guccione

**Affiliations:** ^1^Department of Pharmacology, Institute of Clinical Medicine, University of Oslo and Oslo University HospitalOslo, Norway; ^2^K.G. Jebsen Cardiac Research Centre and Center for Heart Failure Research, Faculty of Medicine, University of OsloOslo, Norway; ^3^Section of Biochemistry and Molecular Biology, Department of Biological, Geological and Environmental Sciences, University of CataniaCatania, Italy; ^4^Department of Drug Sciences, University of CataniaCatania, Italy; ^5^Department of Chemical Sciences, University of CataniaCatania, Italy; ^6^Section of Catania, National Institute of Biostructures and BiosystemsCatania, Italy

**Keywords:** homology modeling, mutagenesis, molecular dynamics, docking, G protein, adenylyl cyclase

## Abstract

The human 5-HT_7_ receptor is expressed in both the central nervous system and peripheral tissues and is a potential drug target in behavioral and psychiatric disorders. We examined molecular determinants of ligand binding and G protein activation by the human 5-HT_7(a)_ receptor. The role of several key residues in the 7th transmembrane domain (TMD) and helix 8 were elucidated combining *in silico* and experimental mutagenesis. Several single and two double point mutations of the 5-HT_7(a)_ wild type receptor were made (W7.33V, E7.35T, E7.35R, E7.35D, E7.35A, R7.36V, Y7.43A, Y7.43F, Y7.43T, R8.52D, D8.53K; E7.35T-R7.36V, R8.52D-D8.53K), and their effects upon ligand binding were assessed by radioligand binding using a potent agonist (5-CT) and a potent antagonist (SB269970). In addition, the ability of the mutated 5-HT_7(a)_ receptors to activate G protein after 5-HT-stimulation was determined through activation of adenylyl cyclase. *In silico* investigation on mutated receptors substantiated the predicted importance of TM7 and showed critical roles of residues E7.35, W7.33, R7.36 and Y7.43 in agonist and antagonist binding and conformational changes of receptor structure affecting adenylyl cyclase activation. Experimental data showed that mutants E7.35T and E7.35R were incapable of ligand binding and adenylyl cyclase activation, consistent with a requirement for a negatively charged residue at this position. The mutant R8.52D was unable to activate adenylyl cyclase, despite unaffected ligand binding, consistent with the R8.52 residue playing an important role in the receptor-G protein interface. The mutants Y7.43A and Y7.43T displayed reduced agonist binding and AC agonist potency, not seen in Y7.43F, consistent with a requirement for an aromatic residue at this position. Knowledge of the molecular interactions important in h5-HT_7_ receptor ligand binding and G protein activation will aid the design of selective h5-HT_7_ receptor ligands for potential pharmacological use.

## Introduction

The 5-HT_7_ receptor is a seven-transmembrane spanning receptor, coupled primarily to the stimulatory G protein (G_s_). It is found in multiple organ systems, such as the cardiovascular system, CNS and digestive tract. In the central nervous system it has been proposed to play a role in the action of antipsychotics and antidepressants and it seems to be involved in regulating circadian rhythms and thermoregulation, learning and memory, as well as rapid eye movement (REM) sleep via the modulation of suprachiasmatic nucleus neurons (Gellynck et al., 2013). 5-HT_7_ receptor agonists have been suggested in treatment of dysfunctional memory in age-related decline and Alzheimer’s disease (Meneses, 2014), as well as treatment of pain, migraine, schizophrenia, anxiety and cognitive disturbances (Gellynck et al., 2013; Gasbarri and Pompili, 2014). A possible involvement in regulation of mood suggests that 5-HT_7_ is a potential target for the treatment of depression (Gellynck et al., 2013). In the periphery the 5-HT_7_ receptor is found primarily in the smooth muscle cells of blood vessels (Ullmer et al., 1995), and in the gastrointestinal tract, where it mediates relaxation of the ileum and stomach (Prins et al., 1999), and was recently shown to be important in inflammation (Guseva et al., 2014).

Three human 5-HT_7_ receptor splice variants (h5-HT_7(a)_, h5-HT_7(b)_, h5-HT_7(d)_) have been identified that are structurally identical except in their predicted intracellular carboxyl terminal (C-terminal) tail. They have indistinguishable pharmacological properties and similar abilities to stimulate adenylyl cyclase, indicating that the C-terminal tail does not influence ligand binding or G-protein coupling (Krobert et al., 2001). To facilitate development of selective drugs targeting the h5-HT_7_ receptor, it is necessary to understand the molecular interactions involved in ligand binding to the receptor. Several studies have focused on the molecular interaction of endogenous serotonin with different 5-HT receptor subtypes. Molecular requirements for serotonin binding to its receptor include electrostatic interaction between the receptor and the amino group of the ligand, one hydrogen bond between donor-acceptor site of the receptor and the hydroxyl group of serotonin and finally van der Waals interactions.

Mutagenesis studies of serotonin receptors (Ho et al., 1992; Wang et al., 1993; Boess et al., 1998; Mialet et al., 2000) suggested that the serotonin amino group makes an electrostatic interaction with the carboxylate of the highly conserved aspartate D3.32. This interaction is usually found in biogenic amine receptors, generally involved in binding of agonists/antagonists and in receptor activation. And there is evidence that serotonin hydroxyl groups interact with a donor-acceptor hydrogen bond residue present in transmembrane domain (TMD) V. These interactions seem relevant to only some serotonin receptor subtypes including 5-HT_1A_, 5-HT_2_, 5-HT_6_ and 5-HT_4_ (Mialet et al., 2000). The role of D3.32 was not relevant for antagonist interactions at the 5-HT_1A_ receptor and the role of the hydrogen bond donor residue of the TMDV was not unambiguously identified in either 5-HT_1A_ or 5-HT_2B_ receptors (Wang et al., 2013). These anomalies indicate that the role of individual residues in molecular receptor-ligand interactions vary among different serotonin receptor subtypes.

To determine the critical molecular interactions that mediate ligand binding in the h5-HT_7_ receptor we used an experimental and *in silico* site-directed mutagenesis approach. Using homology modeling of the β_2_-adrenergic receptor (pdb: 2RH1; Cherezov et al., 2007), a 3D model of the h5-HT_7_ receptor was built and stabilized by large scale simulation in membrane bilayers. We focused on several specific amino acids, located in the 7th TMD, on the basis of preliminary modeling considerations, as discussed in the results section. In order to verify the predicted interactions, several h5-HT_7(a)_ receptor mutants were generated, expressed and assessed for ligand binding as well as agonist and antagonist effects on adenylyl cyclase activity. Our results indicated that residues E7.35, R7.36 and Y7.43 are critical for ligand binding, while residue R8.52 plays a key role in G protein activation.

## Methods

### Mutation Strategy and Mutagenesis

A preliminary model comparison between the β_2_-adrenergic and h5-HT_7(a)_ receptors was performed and the results showed a strong sequence homology, especially at the level of specific transmembrane helices. Docking of 5-HT into the orthosteric binding cavity of our 5-HT_7_ model revealed a critical role of residues W7.33, E7.35, Y7.43, R8.53 and D8.54, where some were similar to that previously reported for the 5-HT_7_ receptor based on ligand docking (Kołaczkowski et al., 2006), and for 5-HT_1B_ and 5-HT_2B_ receptors based on crystallization (Wang et al., 2013). To understand how specific amino acids were involved in the binding process, determination of the 5-HT_7_ single point mutations was based on the chemical-physical characteristics of the specific amino acid with the objective to change the amino acid charges. In addition, point mutations were made to remove the aromatic groups to determine their importance for the interaction with the specific ligands. By site-directed mutagenesis we generated several clones expressing h5-HT_7(a)_ receptors with single or double mutated amino acids.

### Molecular Modeling

Molecular dynamics (MD) simulations and docking studies were carried out using an Intel Core i7 processor, 16 GB RAM, operating under Linux/Ubuntu 10.04. The homology model of the h5-HT_7_ receptor was kindly supplied by Prof. Ingebrigt Sylte’s group (unpublished data) and built from the crystal structure of the β_2_-adrenergic receptor (pdb: 2RH1; Cherezov et al., 2007) by SwissModel server.^1^ Conformations of residues that differ between the 5-HT_7_ and β_2_-adrenergic receptors have been optimized using the RefineModel macro of ICM (Abagyan and Totrov, 1994).

The homology model features a disulfide bridge between TMH3 and extracellular loop 2 (ECL2) which are in accordance with the template structure. All the wild type and mutants considered (W7.33V, E7.35T, E7.35R, E7.35D, E7.35A, R7.36V, Y7.43A, R8.52D, D8.53K and R8.52D-D8.53K) were embedded in a bilayer of POPC (palmitoyl-oleyl-phosphatidyl choline; 100 × 100 Å) and solvated with pre-equilibrated water molecules in the three-dimensional space (box of water) of 130 × 130 × 108 Å. The counterions (Na^+^ and Cl^−^) were placed in the proximity of the regions of the protein surface to mimic an ionic strength of 0.15 mM. Thirty-five nanoseconds of MD simulations were carried out for all the considered systems using the NAMD2 software, version 2.9 (Phillips et al., 2005) with the CHARMM27 force field where all atoms are explicitly represented and water is characterized by the TIP3P model with a dielectric constant of 1 (ε) (Jorgensen et al., 1983). All systems were energy-minimized (conjugate gradient) then gradually heated up to 300 K with a 2-fs time step and equilibrated with a 300 K thermal bath for 400 ps. The velocities were reassigned every 2 ps to achieve complete stability (Berendsen et al., 1984). Production runs were performed at 300 K. The SHAKE algorithm with a tolerance of 1 × 10^−8^ Å was used to fix the length of the covalent hydrogen bonds (Ryckaert et al., 1977). Non-covalent interactions were calculated at each step. To avoid edge effects and treat long-range electrostatic interactions, periodic boundary conditions and the particle-mesh-Ewald algorithm with a grid size of 130 × 130 × 108 Å (Essmann et al., 1995) respectively, were applied to all of the simulation steps. Non-bonded short-range interactions were treated by a cutoff value of 10 Å. After minimizing the protein, the helices were first equilibrated in water with constraints on the cytosolic side. Next, MD of the helices with added loops and disulphide bonds was performed. Finally, MD of the unconstrained domain embedded in a bilayer POPC was carried out.

The ten mutants of 5-HT_7_ were constructed based upon the optimized model obtained as described above, adopting the mutate plugin of VMD 1.91. The mutants studied were W7.33V, E7.35A, E7.35D, E7.35R, E7.35T, R7.36V, E7.35T-R7.36V, Y7.43A, Y7.43T, Y7.43F, R8.52D, D8.53K, and R8.52D-D8.53K. 30 ns of MDs calculation for each mutant was performed, using computational resources granted from the supercomputing HPC-CINECA and UK-NSC. The predicted trajectories were analyzed using the VMD 1.91 software (Humphrey et al., 1996). The average structures as extracted from the last xyz atomic coordinates (50 ps for each MD simulation) were used as input file for docking.

Semiflexible docking was carried out by the molecular docking algorithm MolDock Optimizer and the scoring function MolDock [GRID] as implemented in the Molegro Virtual Docker software, version 6 (Thomsen and Christensen, 2006). Only torsion angles in the side chains were modifed during the minimization; all other properties, including bond lengths and backbone atom positions, were held fixed, and a new receptor conformation was generated for each pose after each docking calculation. The 10 runs for each molecule were carried out with a population size of 50, maximum iteration of 2000, scaling factor of 0.50. 5-carboxamidotryptamine (5-CT) and SB269970 structures were built and minimized by the software SYBYL-X 1.3.^2^

### Site-Directed Mutagenesis

All mutant h5-HT_7(a)_ sequences used in this work were obtained by site-directed mutagenesis of the coding region of the human h5-HT_7(a)_ sequence cloned in pcDNA3.1 vector (Krobert et al., 2001). We used the QuikChange® Site-Directed Mutagenesis kit containing a *Pfu*Turbo DNA polymerase (Agilent). All mutated sequences were confirmed by DNA sequencing. Using this strategy we generated a set of h5-HT_7(a)_ clones carrying a single mutation at the following positions: W7.33V, E7.35T, E7.35R, E7.35D, E7.35A, R7.36V, Y7.43A, Y7.43F, Y7.43T, R8.52D, D8.53K. We also generated h5-HT_7(a)_ clones with the double mutations E7.35T-R7.36V and R8.52D-D8.53K.

### Expression of Wild-Type and Mutant Human 5-HT_7(a)_ Receptors and Cell Culture

The human 5-HT_7(a)_ receptor mutants obtained were expressed in QBI-HEK293 cells which were grown in Dulbecco’s modified Eagle’s medium (DMEM; GIBCO) with 10% fetal bovine serum (BioWhittaker) and penicillin (100 U/ml) and streptomycin (100 μg/ml). Cells were transiently transfected with wild-type and mutated plasmid DNA using LIPOFECTAMINE™—LTX reagent (Invitrogen™) and 7.5 μg DNA per 150 mm dish and a serum-free medium (ULTRAculture, BioWhittaker; because serum contains high concentration of serotonin) supplemented with L-glutamine (2 mM), penicillin (10000 U/ml) and streptomycin (10000 U/ml).

### Membrane Preparation

Membrane preparations from transiently transfected QBI-HEK293 cells containing the mutated receptors were prepared 48 h after transfection as described previously (Krobert et al., 2001).

### Binding Assays

The receptor expression level of QBI-HEK293 cells expressing mutated h5-HT_7(a)_ was determined by radioligand binding. Binding assays were performed in 96-well, round-bottom microtiter plates with total reaction volume of 50 μl, containing the indicated concentration of ligand ([^3^H]5-CT (serotonin agonist) or [^3^H]SB269970 (serotonin antagonist)) as previously described (Krobert et al., 2001). Specific binding was defined as the difference between total binding and non-specific binding (obtained in the presence of 10 μM 5-HT). G-protein-coupled receptors may exist in different conformations, such as low and high affinity states. In the presence of excess GTP most receptors will exist in the low affinity state. To avoid bias due to unpredictable ratios between the two affinity states, GTP (100 μM) was included in the assay mix.

### Adenylyl Cyclase Assays

Adenylyl cyclase activity was measured by determining conversion of [α-^32^P]ATP to [^32^P]cAMP in membrane preparations as previously described (Krobert et al., 2001). Briefly, adenylyl cyclase activities were measured on 10 μl aliquots in a final volume of 50 μl in the presence of 0.1 mM [α-^32^P]ATP, 4 mM MgCl_2_, 20 μM GTP, 1 mM EDTA, 1 mM [^3^H]cAMP, 1 mM 3-isobutyl-1-methylxanthine (IBMX; Sigma), a nucleoside triphosphate regenerating system and additives (FSK (100 μM), 5-CT, 5-HT and SB269970). The samples were incubated for 20 min at 32°C. Cyclic AMP formed was quantified by the double column chromatography system on Dowex 50 cation exchanger and on neutral aluminium oxide (Alumina) columns. The recoveries of each sample through the columns are monitored by adding [^3^H]cAMP to the assay mix, thus eliminating individual differences between the columns.

### Binding and Adenylyl Cyclase Data Analysis

Binding and adenylyl cyclase data were analyzed by non-linear regression using Microsoft Excel 2007 with the Solver add-in, as described (Krobert et al., 2001).

### Western Blot

Lysates of membrane preparations were separated by SDS-PAGE and electroblotted as described (Norum et al., 2005). The membranes were incubated overnight at 4°C with a 1:200 dilution (v/v) of rabbit anti-5-HT_7_R (Oncogene Research Products, Boston, MA) in PBS containing 5% (w/v) non-fat dry milk and 0.05% (v/v) Tween 20. Thereafter, the blots were incubated with a 1:5000 dilution (v/v) of HRP-linked anti-rabbit IgG (Amersham ECL™-HRP Linked Secondary Antibodies, GE Healthcare). The immobilized HRP-conjugated secondary antibodies were visualized with the LumiGLO Chemiluminescent Substrate (KPL, Inc.) and visualized with a UCP Sensicam (UVP Inc., CA, USA).

## Results

### Modeling

**Figure 1:**
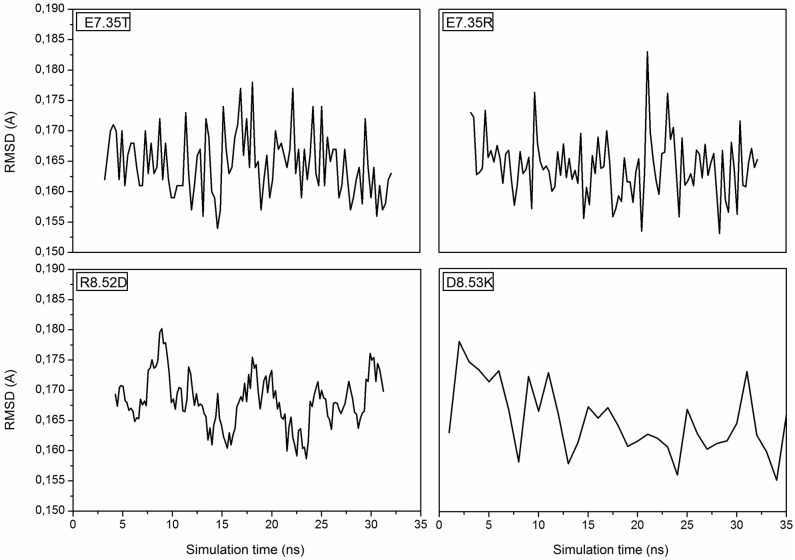
**Root Mean Square Deviation (RMSD) is referring to the backbone of the four mutants E7.35T, E7.35R, R8.52D and D8.53K**.

MD simulations of the mutants of 5-HT_7_ were performed in order to obtain a reliable structure for the subsequent docking calculation. After about 30 ns of equilibration root mean square deviation (RMSD) profiles were analyzed for all mutants to verify the backbone stability and perform docking analysis. All RMSD curves are steady along the whole equilibration phase and no noteworthy oscillations were observed, demonstrating that all structures were stabilized and equilibrated by 35 ns of MD. Figure 1 shows the trend of RMSD for backbone atoms of the mutated receptors E7.35T, E7.35R, R8.52D and D8.53K. All analyzed models showed a high structural rigidity; for all models the RMSD was about 0.165 Å.

**Figure 2:**
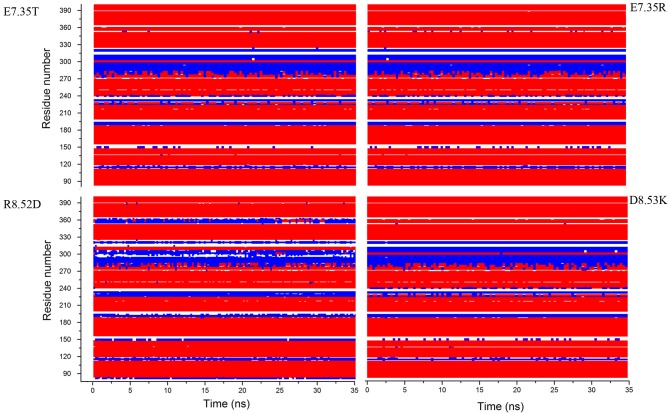
**Secondary structure for the mutants E7.35T, E7.35R, R8.52D and D8.53K calculated over the molecular dynamic (MD) trajectory**. Red represents helix conformation, blue represents turn conformation, white represents random coil conformation.

**Figure 3:**
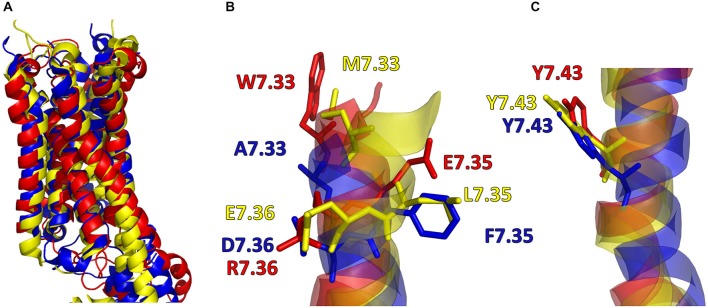
**(A)** Superimposition of 5-HT_7_ after 30 ns of equilibration (red) with 5-HT_1B_ (blue) (Wang et al., 2013) and 5-HT_2B_ (yellow) (Wacker et al., 2013). **(B,C)** Main chain of the three receptors represented as stick and aminoacid residues as lines.

In addition, in Figure 2, the secondary structures of the models were plotted over time, in order to verify that the predicted secondary structure does not change significantly. The backbone of the seven trans-membrane domains seems to be stable in helix conformation during the simulation. On the other hand, residues of the extracellular loops appear to be more flexible, and they do not have a stable conformation. At the end of 30 ns of equilibration, our model consists of seven transmembrane helices (Figure 2) and a large cavity defined by the helices III, V, VI and VII, in accordance with Wang et al. (1993). Comparison of our 5-HT_7_ model with the X-ray structure of 5-HT_1B_ (4IAQ) (Wang et al., 2013) and 5-HT_2B_ (4IB4) (Wacker et al., 2013) receptors, confirms that the main folding pattern of our model appears to be consistent with experimental data, as reported in panel A of Figure 3. While the three structures here compared have slightly different primary sequences (e.g., T6.46 in 5-HT_7_, M6.46 in 5-HT_2B_ and A6.46 in 5-HT_1B_), the same region with different amino acids has the same spatial orientation of the side chains, as evidenced in Figure 3B. Particularly, glutamic acid is conserved in all the models, along with its orientation. Moreover, in our model a formation of a hydrogen bond between T7.35 and R6.58 is observed, as discussed later in the discussion section, which should play a role in ligand binding. All the data indicate that our models appear good candidates for further study.

Docking results have been summarized in Table 1. All the mutants are predicted to bind both the agonist 5-CT and the antagonist SB269970. Tables 2, 3 list the favorable and unfavorable interactions of the two reference molecules with the wild type and mutant receptors. Overall, the antagonist (SB269970) forms a higher number of positive interactions and no unfavorable steric interactions that allow the formation of more stable complex (more negative docking score) than the agonist. Mutation of E7.35 to Asp, Thr and Arg were predicted to produce better binding compared to the wild type protein, while mutation of E7.35 to Ala was predicted to maintain high binding capacity. On the contrary, mutation of R8.52 to Asp was predicted to produce weaker binding. Only mutant D8.53K was predicted to show slightly lower binding. In this case the increased steric hindrance of the mutations plays a key role in binding properties.

**Table 1:** **MolDock score (a.u.) from docking analysis of wild type and mutant receptors with 5-CT and SB269970**.

	5-CT	SB269970
**5-HT_7(a)_**	−93	−132
**W7.33V**	−95	−127
**E7.35T**	−102	−129
**E7.35R**	−103	−151
**E7.35D**	−98	−127
**E7.35A**	−100	−124
**R7.36V**	−109	−144
**Y7.43A**	−100	−126
**R8.52D**	−92	−111
**D8.53K**	−89	−119
**R8.52D-D8.53K**	−114	−119

**Table 2:** **List of favorable and unfavorable interactions after docking between SB269970 and the target molecules**.

Protein	H bond interactions	Hydrophobic interactions	Favorable steric interaction
Wild type	Cys231, Ile233, E7.35, T2.64, R7.36	I3.29, Leu232, Leu236, L7.39, F2.60, F3.28 (pi stacking), V2.61, Val230	R6.58, S6.55
E7.35A	T4.57, S5.42, Gln235	A5.46, I4.56, Ile233, L4.61, F5.47, F6.52, P4.60, T3.37, Trp221, V3.33	D3.32, C3.36, T5.39, T5.43
E7.35D	D3.32, S6.55	I3.29, Ile233, Leu232, L4.61, F3.28, F6.51, F6.52 (pi-stacking), T5.43 (t-stacking), W6.48, V3.33	R6.58, C3.36, Gln235
E7.35R	W6.48, Y7.43	A5.46, I3.29, Ile233, Leu232, L7.39, F3.28, F6.44, F6.51, F6.52, W6.48, Y7.43, V2.53, V3.33	D3.32, C3.35, C3.36, Cys231
E7.35T	I4.56, T5.43	A5.46, I4.56, Ile233, L4.61, L7.39, F5.47, F6.51, F6.52, T3.37, T4.57, T5.39, T5.43, W6.48, V3.33, V5.45	D3.32, C3.36, Gln235, S5.42
R8.52D	C3.36	A5.46, L6.49, L7.39, M3.34, F5.47, F6.44, F6.51, F6.52 (t-stacking), T3.37, T5.43, W6.48 (t-stacking), V3.33	D3.32
V7.33W	C3.36, D3.32	A5.46, I3.29, I3.40, L7.39, F5.47, F6.51, F6.52, T3.37, T4.57, T5.43, W6.48 (pi-stacking), Y7.43, V2.53, V3.33	S5.42, S6.55
Y7.43A	S6.55	I3.29, Ile233, Leu232, L7.39, F3.28, F6.51, F6.52 (pi-stacking), W6.48 (t-stacking), V3.33	R6.58, D3.32, C3.36, S6.55
R7.36V	G7.42	A2.49, G7.42, L7.39, L7.41, F5.47, F6.51, F7.38, W6.48, W7.40, Y7.43, V2.53	D3.32, C3.35, C3.36, C6.47, S3.39, S7.46
D8.53K	F6.52	A5.46, Ile233, I5.40, L4.61, F4.62, F6.52, P4.60, P6.59, Y5.38, Y5.48, V3.33	Gln223, Gln235, S5.42, S6.55, T4.57, T5.39, T5.43, T6.56
R8.52D-D8.53K	T4.57, T3.37	A5.46, I3.40, I4.56, L7.39, F6.51, F5.47, F6.52, T5.39, W6.48, Y7.43, V3.33	D3.32, C3.36, S5.42, T3.37, T4.57, T5.43

*AAs which are part of the helices are numbered according to Ballesteros-Weinstein nomenclature (Ballesteros and Weinstein, 1995)*.

**Table 3:** **List of favorable and unfavorable interactions after docking between 5-CT and the target molecules**.

Protein	H bond interactions	Hydrophobic interactions	Favorable steric interactions
Wildtype^(1)^	I3.29, D3.32, Y7.43, Cys231	A3.30, Ile233, Leu232, L7.39, F3.28 (stacking), V2.61, V2.57, V3.33	R6.58, E7.35
E7.35A	I4.56, V3.33, Gln235, T3.37	A5.46, Ile233, L4.61, P4.60, F6.52, T5.43, Trp221, Y5.38, V3.33, V5.45	S5.42, T4.57
E7.35D	S6.55, Cys231, I3.29	A3.30, Leu232, Ile233, F3.28 (t-stacking), F6.51, V3.33, Val230	R6.58, D3.32, Gln235
E7.35R	D3.32, Y7.43	A3.30, I3.29, Ile233, Leu232, L7.39, F3.28, F6.51, F6.52, W6.48, Y7.43, V3.33	Cys231
E7.35T	A5.46, T5.43, Gln235, D3.32	A5.44, A5.46, I3.40, M3.34, F5.47, F6.52 (t-stacking), V3.33	C3.36, S5.42, T3.37, T5.39
R8.52D	T4.57, I3.29	A3.30, I3.29, Ile233, L4.58, L4.61, P4.60, S5.42, Trp221, Y5.38, V3.33, Val225	Asn224, Gln223, Gln235, T4.57
V7.33W	D3.32, T5.39, T5.43, T3.37	A5.46, I3.40, L4.61, F5.47, F6.52, Y5.38, V3.33	C3.36, Gln235, S5.42, T3.37, T4.57, T5.39, T5.43
Y7.43A	S6.55, T5.43	A5.46, Ile233, F5.47, F6.52, P6.59, Y5.38 (t-stacking), V3.33	Gln235, Ser234, S5.42, S6.55, T4.57, T5.39, T6.56
R7.36V^(2)^	L7.39, W6.48, G7.42, S7.46	G7.42, L6.49, L7.39, L7.41, F5.47, F6.51, F6.52, F7.38, W6.48, W7.40, Y7.43 (t-stacking), V2.53	C3.36, C6.47, D2.50(electro-static interaction), S7.46
D8.53K	Gln235, Ser234	Ala222, Gly220, I3.29, Ile233, L4.61, Leu232, F4.62, P4.60, Trp221, Y5.38, Val225	Asn224, Gln223, Gln235, Ser234, T5.39
R8.52D-D8.53K	A5.46, T4.57, I4.56, S5.42, T3.37	A5.46, I3.40, I4.56, F5.47, F6.52, V3.33	C3.36, S5.42, T3.37, T4.57, T5.39, T5.43

*AAs which are part of the helices are numbered according to Ballesteros-Weinstein nomenclature (Ballesteros and Weinstein, 1995). ^(1)^ Unfavorable electrostatic interactions with R6.58 and Lys229. ^(2)^ Unfavorable electrostatic interactions with R6.58. Favorable electrostatic interaction with D2.50*.

### Characterization of Human 5-HT_7(a)_ Mutants

#### Ligand Binding

The mutant receptors were examined by comparing the binding properties and ability to activate adenylyl cyclase (cyclic AMP production), using transiently transfected QBI-HEK293 cells. The affinities of the agonist (5-CT) and the antagonist (SB269970) determined from saturation binding experiments with up to 3 nM of [^3^H]5-CT and up to 2.5 nM [^3^H]SB269970 varied from being not modified, reduced or, in some mutants, no specific binding was detected. The receptor density of all the analyzed mutants was only slightly reduced in a subset of mutants, so receptor density is unlikely to account for changes in affinity (shown in Table 4). In addition, the ability to activate AC was either unmodified or reduced in the mutated receptors compared to wild-type (Table 5). Most importantly, the mutagenesis studies revealed critical roles of residues E7.35, R7.36 and Y7.43 in ligand binding and R8.52 in activation of AC.

**Table 4:** **Binding properties of wild-type and mutant 5-HT_7(a)_ receptors expressed in QBI-HEK293 cells**.

Mutant	[^3^H]5-CT			[^3^H]SB269970		
	pK_d_		B_max_ (pmol/mg protein)	pK_d_		B_max_ (pmol/mg protein)
**5-HT_7(a)_**	9.51 ± 0.05		2.00 ± 0.27	9.51 ± 0.57		1.79 ± 0.27
**W7.33V**	9.57 ± 0.11		1.94 ± 0.09	9.30 ± 0.07		1.92 ± 0.21
**E7.35T**		No specific binding			No specific binding	
**E7.35R**		No specific binding			No specific binding	
**E7.35D**	9.17 ± 0.09		2.14 ± 1.01	9.50 ± 0.06		2.08 ± 0.63
**E7.35A**	8.74 ± 0.32 *		1.14 ± 0.03	9.41 ± 0.01		1.36 ± 0.15
**E7.35T-R7.36V**		No specific binding			No specific binding	
**R7.36V**	8.17 ± 0.54 *		0.66 ± 0.37 ^†^	9.77 ± 0.03		0.26 ± 0.06 ^†^
**Y7.43A**	8.55 ± 0.29 *		0.21 ± 0.09 ^†^	8.98 ± 0.34		0.27 ± 0.16 ^†^
**Y7.43T**	8.55		0.21	8.56		0.41
**Y7.43F**	9.21		2.78	9.62		2.52
**R8.52D**	9.17 ± 0.04		0.53 ± 0.06 ^†^	9.09 ± 0.01		0.46 ± 0.07 ^†^
**D8.53K**	9.30 ± 0.07		1.22 ± 0.15 ^†^	9.25 ± 0.09		1.09 ± 0.04
**R8.52D-D8.53K**	9.57 ± 0.09		1.20 ± 0.07	9.53 ± 0.10		1.19 ± 0.07

Wild-type (5-HT_7(a)_) and mutant receptors were transiently transfected in QBI-HEK293 cells. Membranes were subjected to saturation binding analysis using [^3^H]5-CT and [^3^H]-SB269970 as selective ligands. Affinity values are presented as pK_d_ values (−log Kd). Data shown are average ± SEM of 3–5 experiments, except for Y7.43T and Y7.43F (*n* = 1). * − *P* < 0.05 One-way ANOVA with Dunnett’s multiple comparisons test vs. wild-type. ^†^ − *P* < 0.05 One-way ANOVA with Dunnett’s multiple comparisons test vs. wild-type within experiments.

**Table 5:** **Activation of adenylyl cyclase (AC) by the agonists 5-CT and 5-HT and inhibition of 10 μM 5-HT-stimulated AC by the antagonist SB269970**.

Mutant	pEC_50_		pK_i_
	5-CT	5-HT	SB269970
**5-HT_7(a)_**	7.82 ± 0.07	7.06 ± 0.03	8.90 ± 0.10
**W7.33V**	7.99 ± 0.16	7.27 ± 0.12	8.76 ± 0.31
**E7.35T**	N.D.	N.D.	N.D.
**E7.35R**	N.D.	N.D.	N.D.
**E7.35D**	7.67	6.85	9.12
**E7.35A**	7.39	6.48	8.77
**E7.35T-R7.36V**	N.D.	N.D.	N.D.
**R7.36V**	7.89	6.69	9.67
**Y7.43A**	7.62 ± 0.28	6.76 ± 0.04 *	8.13 ± 0.03 *
**Y7.43T**	7.45	5.98	7.28
**Y7.43F**	8.15	7.23	8.98
**R8.52D**	N.D.	N.D.	N.D.
**D8.53K**	7.69 ± 0.06	6.81 ± 0.10	8.59 ± 0.13
**R8.52D-D8.53K**	6.91 ± 0.07	6.28 ± 0.08	9.15 ± 0.16

Potency values are presented as pEC_50_ (−log EC_50_) values for agonists and pK_i_ (−log K_i_) values for antagonists, calculated by the method of Cheng and Prusoff (1973). Data shown are average ± SEM of 1–8 experiments. N.D., no AC activity detected. *p < 0.05 One-way ANOVA with Dunnett’s multiple comparisons test vs. wild-type.

Initially, E7.35 was mutated to Thr to remove the negative charge in this part of the receptor and this new receptor showed a complete lack of ability to bind either agonist or antagonist. To understand which kind of interaction is critical in this part of the receptor for the ligand-receptor-interaction, other mutations were performed at E7.35: (1) to Asp (E7.35D) to understand the importance of amino acid charge; (2) to Ala (E7.35A) to test if the length of the side chain is important for ligand binding; and (3) to Arg (E7.35R) to determine if there are electrostatic interactions involved. These new mutants (E7.35D, E7.35A, E7.35R) were analyzed as above in the binding assays. While the E7.35R mutant receptor was completely unable to bind either agonist or antagonist, the E7.35A mutant displayed reduced binding affinity for agonist, but not for antagonist, and the E7.35D mutation did not change the ability of the receptor to bind any ligands (Table 4). Mutation of the neighboring residue, R7.36V, resulted in decreased binding affinity for agonist only and a reduced receptor expression. The double mutant E7.35T-R7.36V was unable to bind any ligands.

The mutation R8.52D, where the positive charge was replaced by a negative one, did not alter the affinity for 5-CT or SB269970 (but the receptor expression was significantly reduced), neither did the mutation D8.53K nor the double mutant R8.52D-D8.53K. Another TMH7 mutation evaluated was Y7.43A, where the aromatic group of residue Y7.43 was replaced with the non-polar Ala residue. This mutation caused a significant reduction in agonist binding affinity and a possible reduction in antagonist binding affinity, in addition to a reduction in receptor expression. To determine if the aromatic group or OH-group was involved in the changes elicited by mutation Y7.43A, mutant receptors Y7.43F and Y7.43T were constructed. Preliminary data indicate that Y7.43T showed a reduction in ligand potency and receptor expression, whereas Y7.43F was without effect upon ligand binding.

#### Effect upon Adenylyl Cyclase Activation

**Figure 4:**
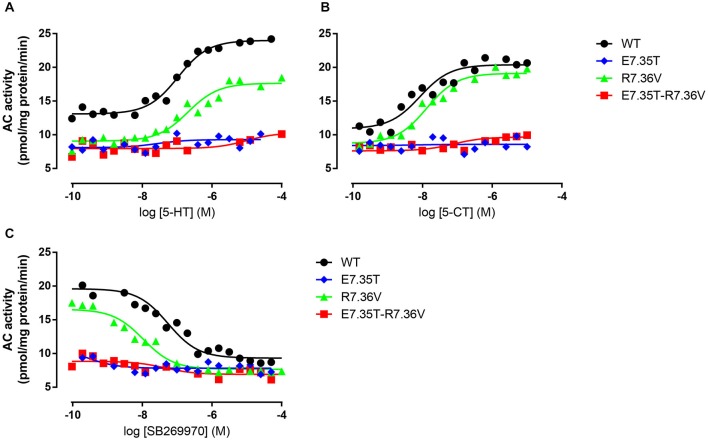
**Ability of mutant receptors to activate adenylyl cyclase**. Adenylyl cyclase (AC) activity in response to increasing concentrations of 5-HT **(A)** or 5-CT **(B)** in membranes from QBI-HEK293 cells transiently expressing the wild-type (WT) or indicated mutant receptors. In **(C)** the ability of increasing concentrations of SB269970 to antagonize 10 μM 5-HT was determined. AC activity was measured as described under *Materials and Methods*, and the data shown are representative of those obtained from 1–3 independent experiments.

We next examined the effect of the different mutations within TMH7 of the h5-HT_7(a)_ receptor on the transductional response (G protein activation) by measuring their ability to activate adenylyl cyclase (AC). As shown in Table 5 and Figure 4, the W7.33V, E7.35D, R7.36V, Y7.43A, and D8.53K mutated receptors stimulated AC activity with the same potency as the wild-type receptor. A possible higher affinity for the antagonist SB269970 of the R7.36V mutant receptor measured in AC assays (Figure 4; Table 5) was not supported by binding data (Table 4) and therefore not investigated further. Particularly interesting was the finding that the R8.52D mutant which displayed unaltered binding affinity for both agonist and antagonist, was not able to activate AC constitutively (no effect of SB269970), or by either of the agonists 5-CT or 5-HT (Table 5). In contrast, the Y7.43A mutant displayed a lower binding affinity for 5-CT, a lower potency on AC for 5-HT and SB269970, but no change in potency to activate AC for 5-CT (Table 5). In line with the lack of binding, the mutants E7.35T and E7.35R showed no ability to activate AC, whereas the conservative mutation E7.35D showed no change in AC activation and the E7.35A mutant activated AC with reduced potency.

#### Lack of Binding not due to Lack of Receptor Expression

To determine if the complete inability of the E7.35T and E7.35R mutants to bind the radioligands [^3^H]5-CT and [^3^H]SB269970 resulted from the absence of receptor expression, we determined if these receptors were in fact expressed by subjecting membrane preparations to SDS-PAGE and determining 5-HT_7_ expression using a polyclonal antibody directed against the N-terminus of the h5-HT_7_ receptor. In those receptors analyzed (even in mutants with reduced or absent radioligand binding), a protein band of about ~50 kDa, corresponding with the size of the h5-HT_7_ receptor, was detected, whereas in non-transfected control cells no band was detected (data not shown). These data indicate that the absence of ligand binding of the various mutant receptors does not result from a lack of receptor expression despite displaying an absence of radioligand binding.

## Discussion

GPCRs are challenging targets in drug design (Overington et al., 2006). Despite the recent surge of GPCR structural data, structures have only been determined for a minor fraction of GPCRs. Therefore, *in silico* tools are key to obtain structural information to integrate and rationalize the design of experimental studies of receptor-ligand interactions. Homology modeling has been successfully applied for several different GPCRs (see Sandal et al., 2013 and references therein). Combining *in silico* with experimental tools can lead to accurate structural characterization and accelerate drug design. In this work, the mechanism of molecular recognition between the h5-HT_7_ receptor and ligands was investigated through *in silico* molecular docking and well-established *in vitro* experimental approaches.

The modeling data predicted that the residue E7.35 of the wild type was relevant for ligand binding. In particular, this residue located in the TMH7 is involved in the formation of a three-member salt bridge with R7.36 and R6.58. Therefore, we focused our attention on this residue and performed single point mutagenesis in order to evaluate the effect of specific amino acids on the binding affinity of the receptor. The chosen mutants E7.35T, E7.35R, E7.35A, E7.35D were created by site-directed mutagenesis and analyzed with radioligand binding assays and the ability to activate AC.

**Figure 5:**
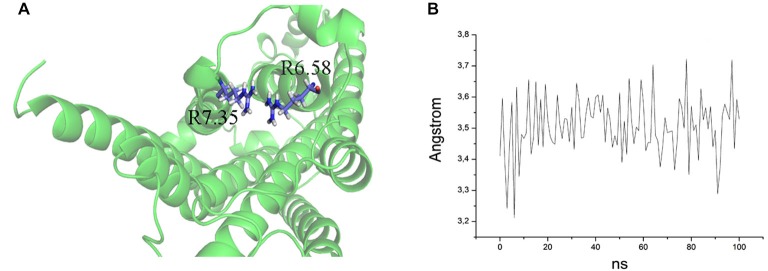
**(A)** Overlap of R7.35 and R6.58 in the mutant E7.35R. The backbone is shown in cartoon representation and the residues are indicated in stick format. **(B)** Distance between the two guanidine groups of R7.35 and R6.58 measured over the MD trajectory.

**Figure 6:**
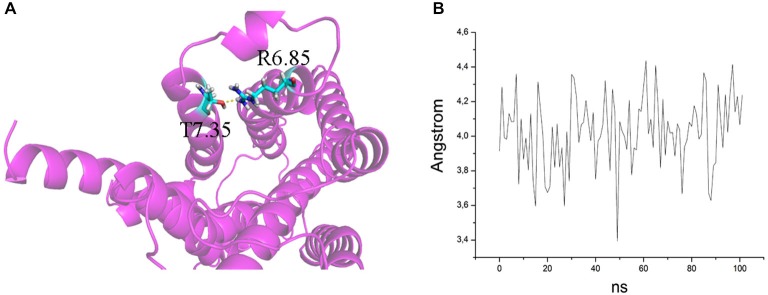
**(A)** H bond between T7.35 and R6.58 in the mutant E7.35T. The backbone is shown in cartoon representation and the residues are indicated in stick format. **(B)** Distance between the oxygen atom of T7.35 and hydrogen aminic atom of R6.58 measured over the MD trajectory.

The ability to bind both the agonist (5-CT) and antagonist (SB269970) radioligand was completely abolished in the E7.35T mutant, despite expression of the mutant receptor as determined by Western analysis. An analysis of the MD trajectory suggests that the inability to activate the receptor may result from the formation of a H bond between T7.35 and R6.58 in the E7.35T mutant. Possibly, this H bond acts as a locked gate preventing the entry of the ligands to the binding cavity (Figure 5). Likewise, no specific binding of either agonist or antagonist ligands was observed in the mutant E7.35R. The MD analysis of E7.35R suggests that the guanidine side chain of R7.35 in the E7.35R mutant moves towards the guanidine side chain of R6.58 blocking the entrance to the orthosteric pocket of the protein (Figure 6). Consistent with the lack of binding, the E7.35T and E7.35R mutant receptors were unable to activate adenylyl cyclase activity (Figure 4; Table 5). Not surprisingly, the conservatively changed mutant E7.35D receptor, maintaining the charged residue, behaved essentially as the WT receptor both regarding binding and AC activation, consistent with the important role of the electrostatic interactions with this residue. Also consistent with this was the finding that the E7.35A mutated receptor, replacing Glu with the nonpolar Ala, showed reduced binding affinity to both agonist (5-CT) and antagonist (SB269970) radioligand (Table 4), and was still able to activate AC, but with reduced potency in line with the reduced binding affinity (Table 5).

The MD analysis showed that another residue was important in the interaction with the ligands: R7.36. The Arg was changed to Val (R7.36V) and the mutation reduced the affinities of agonist (5-CT), but not for antagonist (SB269970) radioligand (Table 4). But the mutant receptor displayed 5-CT-stimulated AC activity with essentially the same properties as the wild-type receptor (Table 5; Figures 4A–C). MD simulations indicated that the R7.36 is involved in a network of interactions. This residue formed an electrostatic interaction with D2.65, which is partially instable (0.8% of whole simulation), due to the formation, as said before, of a three-member salt bridge R7.36-E7.35-R6.58. Consequently, the extracellular part of TMH7 is constrained by two salt bridges to TMH2 and TMH6.

The release of this ionic lock could be a key step in receptor activation. In order to test this hypothesis, charge-neutralizing mutations of E5.67 and of D3.49 were made in the β_2_-adrenergic receptor (Ballesteros et al., 2001). Experimental data by Ballesteros et al. (2001), together with the high-resolution structure of rhodopsin (Palczewski et al., 2000) suggest that ionic interactions between D/E3.49, R3.50, and E6.30 may constitute a common switch governing the activation of many rhodopsin-like G-protein-coupled receptors. In addition, semi-flexible docking calculations were performed at the binding cavity defined by the residues from the 3rd, 5th, 6th, 7th helices and the ECL2, including an orthosteric pocket embedded in the 7TM core and a long binding pocket close to the extracellular site. The binding mode is in accordance with that recently reported for the crystal structure of the h5-HT_1B_ G-protein-coupled receptor bound to ergotamine or dihydroergotamine, which are accommodated at the orthosteric pocket and an extended binding pocket close to the ECL2, respectively (Wang et al., 2013). The residues belonging to the architecture of this pocket are conserved in 5-HT receptors.

The movements of transmembrane segments (TMs) III and VI at the cytoplasmic side of the membrane play an important role in the activation of G-protein-coupled receptors. There is evidence for the existence of an ionic lock that constrains the relative mobility of the cytoplasmic ends of TM3 and TM6 in the inactive state of the β_2_-adrenergic receptor (Ballesteros et al., 2001). The highly conserved R3.50 at the cytoplasmic end of TM3 interacts both with the adjacent D3.49 and with E6.30 at the cytoplasmic end of TM6. Such a network of ionic interactions has now been directly supported by the high-resolution structure of the inactive state of the β_2_-adrenergic receptor (Dror et al., 2009) and would serve to constrain the receptor in the inactive state.

The R8.52D mutant receptor showed a different behavior: although the receptor displayed normal affinity for ligands and a significantly reduced expression (Table 4), the receptor was unable to activate adenylyl cyclase (Table 5). The MD data showed that this residue pointed toward the water during simulation; its position was at the base of helix VII and near the G protein binding domain. We examined the next residue D8.53 to better understand the importance of this region. We changed the Asp to Lys and found that the affinity of both agonist and antagonist ligands for the D8.53K mutant was similar to that observed for the wild-type receptor. A similar result was obtained in the adenylyl cyclase assays, where the D8.53K mutant receptor activated the enzyme with the same efficiency as the wild-type receptor. However, the D8.53K mutation was able to rescue the ability of the R8.52D-mutated receptor to activate AC, since both 5-CT and 5-HT were able to stimulate adenylyl cyclase activity through the double mutated R8.52D-D8.53K receptor with essentially unchanged potency (Table 5) and only a decrease in the efficacy (not shown) compared with the wild-type receptor. Thus the mutagenesis data on the R8.52 residue might be explained by the R8.52D mutation destroying the receptor-G protein interface, possibly by removing the positive charge.

To better understand which kind of interactions are important to bind the specific ligands, to activate the receptor and the downstream pathway, we analyzed the aromatic residues W7.33 and Y7.43. The W7.33 was replaced with Val and Y7.43 with Ala. The mutated receptor W7.33V didn’t change the affinities for 5-CT and SB269970 ligands and stimulated cAMP production with the same efficiency as the wild-type receptor.

The Y7.43A mutant showed a significantly reduced affinity of agonist binding, whereas the reduction in antagonist binding did not reach significance. Kołaczkowski et al. (2006) reported this amino acid to be involved in formation of hydrogen bond with ligands. The two mutants Y7.43F and Y7.43T were designed to assess if the aromatic or the OH-group is involved in the binding process. Based on preliminary data, only the mutant Y7.43T showed a reduction in the ability to bind both the ligands. No changes were observed for the mutant Y7.43F (Table 4). The data are generally in agreement with that for AC activation and antagonism (Table 5). These experimental data were explained by MD simulations, which pointed out that another highly stable salt bridge is formed between Y7.43 and D2.65 in the TMH2 of the 5-HT_7_ receptor.

The actions of ligands at receptors depend on the affinity for the receptor and the activation of a signaling system, termed efficacy, which is positive for agonists as a result of conformational changes. While some of the above mentioned mutations do not seem to affect ligand recognition, they may still prevent or reduce receptor activation and G protein coupling, illustrating independent contributions of these residues in the WT to stabilizing the bound ligands and/or formation of a ligand-induced active state of the receptor (Osaka et al., 1998; Strange, 2008).

## Conclusion

Based on the above reported findings there seems to be a non-ionic lock between helix III and helix VI in the 5-HT_7_ receptor. However, when the salt-bridge between D3.49 and R3.50 is broken a new one is formed between R3.50 and S2.39. The active and inactive states of the 5-HT_7_ receptor are characterized by D3.49-R3.50 and R3.50 and S2.39 salt bridges, respectively. The mutant R8.52D in helix VIII lacks cyclase activation. This residue points toward the water during simulation; its position is at the base of helix VII and near the G protein binding domain. Thus, the mutagenesis data on this residue might be explained by destruction of the receptor-G protein interface, and we can assume that the charge is important.

## Conflict of Interest Statement

The authors declare that the research was conducted in the absence of any commercial or financial relationships that could be construed as a potential conflict of interest.

## Footnotes

^1^http://swissmodel.expasy.org/

^2^http://www.certara.com
